# Seed Metabolome Analysis of a Transgenic Rice Line Expressing Cholera Toxin B-subunit

**DOI:** 10.1038/s41598-017-04701-w

**Published:** 2017-07-12

**Authors:** Takumi Ogawa, Koji Kashima, Yoshikazu Yuki, Mio Mejima, Shiho Kurokawa, Masaharu Kuroda, Atsushi Okazawa, Hiroshi Kiyono, Daisaku Ohta

**Affiliations:** 1 Laboratory of Cell Metabolism and Function, Division of Molecular Biology and Cell Informatics, Department of Applied Life Sciences, Graduate School of Life and Environmental Sciences, Osaka Prefecture University, Osaka, 599-8531 Japan; 2 Tokyo Mucosal Patches Laboratory, Division of Mucosal Immunology, Department of Microbiology and Immunology, The Institute of Medical Science, The University of Tokyo, Tokyo, 108-8639 Japan; 3 International Research and Development Center for Mucosal Vaccines, The Institute of Medical Science, The University of Tokyo, Tokyo, 108-8639 Japan; 4 Division of Crop Development, NARO Central Region Agricultural Research Center, Niigata, 943-0193 Japan

## Abstract

Plant-based human vaccines have been actively developed in recent years, and rice (*Oryza sativa* L.) is one of the best candidate crops for their production and delivery. By expressing a modified cholera toxin B (CTB) subunit, we previously developed MucoRice-CTB, a rice-based vaccine against cholera, which is caused by infection of the intestine with the bacteria *Vibrio cholerae*. MucoRice-CTB lines have been extensively characterized by whole-genome sequencing and proteome analyses to evaluate the mutation profiles and proteome status, respectively. Here, we report non-targeted metabolomic profiling of the MucoRice-CTB transgenic rice line 51A (MR-CTB51A), MucoRice-RNAi (MR-RNAi), and their non-transgenic parent line by using gas chromatography–time-of-flight mass spectrometry. The levels of several amino acids, organic acids, carbohydrates, lipids, and secondary metabolites were significantly increased in MR-CTB51A compared with the non-transgenic parent line. These metabolomics results complement essential information obtained by genome sequencing and proteomics approaches, thereby contributing to comprehensive understanding of the properties of MucoRice-CTB as a plant-based vaccine.

## Introduction

Development of plant-based vaccines has been attempted in recent years. This technology is based on the introduction of genes that encode antigen proteins into the plant genome. Recombinant antigen protein expressed in transgenic plants is expected to induce specific immune responses when the plant material is consumed^1–4^. Plant-based vaccines have several advantages over traditional vaccines: potentially high levels of recombinant protein production; easily scalable production; and low risk of contamination with human pathogens or toxins^3, 5, 6^. Because the induction of mucosal immune responses is efficient, plant-based oral vaccines are expected to be especially useful against mucosal infections^7, 8^. Rice (*Oryza sativa* L.) is a candidate crop for the development of plant-based vaccines^1–4, 9–11^.

MucoRice-CTB (cholera toxin B subunit) has been developed as a rice-seed-based vaccine against cholera, which is caused by the infection of the intestine with the bacteria *Vibrio cholerae*. MucoRice-CTB was first reported as a cold chain–free oral vaccine developed using an authentic CTB gene^3, 5, 6, 9^. The vaccine has been improved by introducing an Asn-to-Gln substitution at the 4th position of the CTB protein (to eliminate plant-specific glycosylation and thus obtain a uniform CTB protein), by removing the selectable marker cassette, and by introducing an RNA interference (RNAi) cassette (to down-regulate endogenous seed storage proteins)^7–9, 12–14^. MucoRice-CTB is a powder prepared from rice (polished to a ratio of 95%) from the MucoRice-CTB transgenic rice line 51A (MR-CTB51A); it was evaluated in a physician-initiated phase I study with healthy volunteers at the Research Hospital of the Institute of Medical Science, the University of Tokyo^15^. A comparative whole-genome analysis using next-generation sequencing demonstrated that MucoRice-CTB transgenic lines (including MR-CTB51A) and their parental non-transgenic lines (*O*. *sativa L*. cv. Nipponbare [NPB]) were almost identical at the genomic level except for the locus where the transgene was inserted^14, 16^. Two-dimensional fluorescence difference gel electrophoresis and shotgun proteome analyses of MucoRice-CTB brown rice revealed that CTB expression together with RNAi-mediated suppression of seed storage proteins in the endosperm did not up-regulate known rice allergens^17^.

Metabolomics is an analytical approach for unbiased identification and quantification of various metabolite classes in biological materials^18–23^, including genetically engineered organisms^24–27^. Combinations of chromatographic and mass spectrometry (MS) techniques are used for metabolomics studies. Gas chromatography (GC)/MS is a powerful analytical method that separates and detects a wide range of low-molecular-weight metabolites such as amino acids, organic acids, sugars, fatty acids, fatty alcohols, wax esters, and sterols^28–32^.

The aim of this study was to investigate whether CTB expression and RNAi-mediated suppression of endogenous seed storage proteins influence rice seed metabolism. We compared the brown rice seed metabolome of MR-CTB51A (grown hydroponically) with those of the parental NPB line (grown either hydroponically [NPB-HP] or in a paddy field [NPB-PF]) and MucoRice-RNAi (MR-RNAi), a transgenic line with RNAi-mediated suppression of endogenous seed storage proteins^17^. The metabolic profiles differed among MR-CTB51A, MR-RNAi, and the parental NPB lines, especially in the contents of hydrophilic primary metabolites and lipids, although no metabolites unique to these transgenic lines were identified.

## Results

### Detection of Metabolite-candidate Peaks

We investigated the metabolite contents in brown rice extracts from MR-CTB51A, MR-RNAi, NPB-HP, and NPB-PF (Supplementary Table S1). From total ion current (TIC) chromatograms obtained by GC/time-of-flight (TOF)-MS analyses, 198 and 153 ion peaks were listed as metabolite-candidate peaks in the non-polar fractions and the polar fractions, respectively (Supplementary Table S2).

### Multivariate Analyses of Metabolite Profiles of MR-CTB51A, MR-RNAi, and Non-transgenic Counterpart NPB

**Figure 1:**
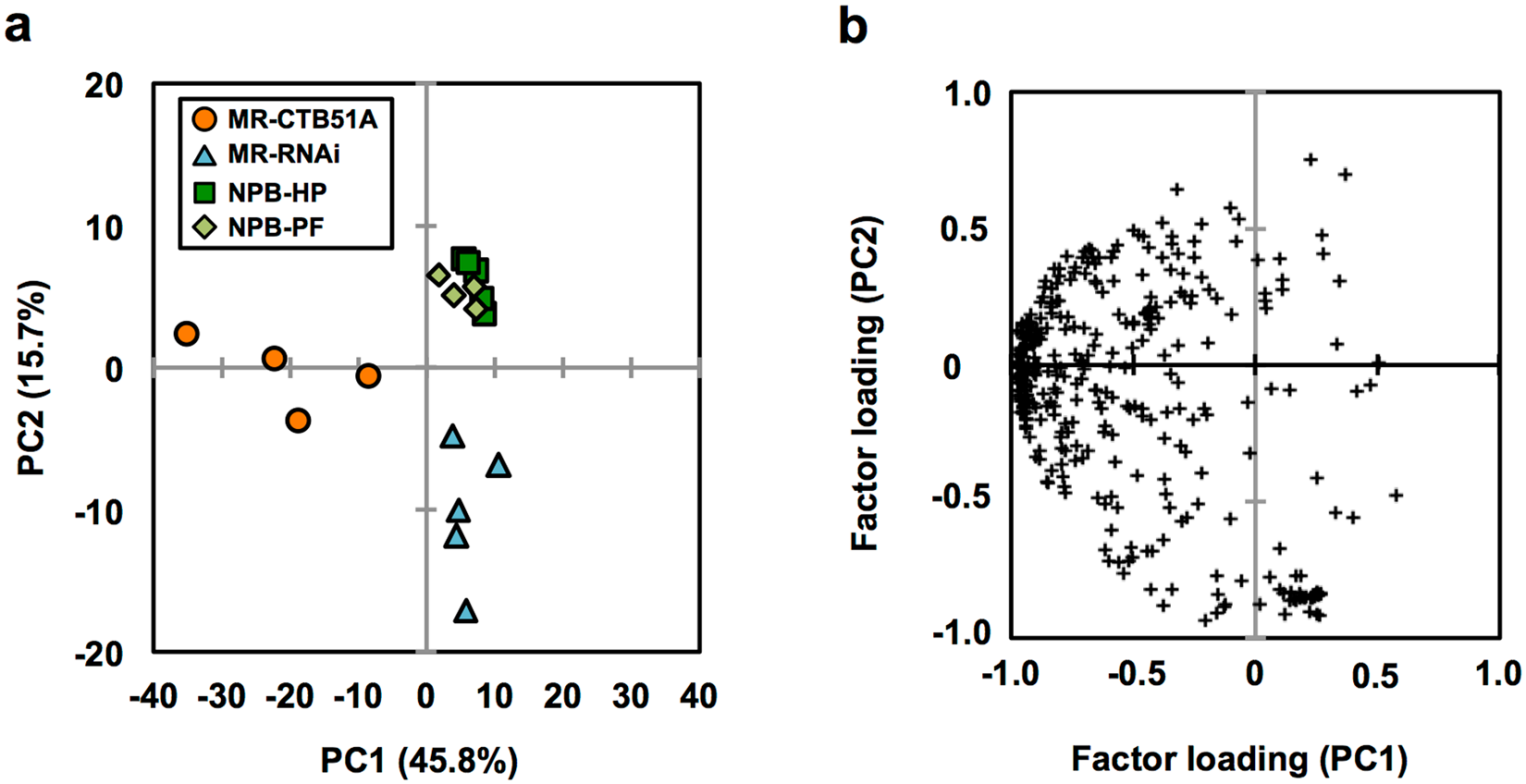
PCA of the metabolic profiling data set. PCA was performed with a metabolite data matrix (351 metabolite-candidate peaks versus samples, relative levels as a variable) obtained from non-polar and polar fractions (Supplementary Table S2). (**a**) Score plot for PC1 vs. PC2. Percentage values in parentheses are the respective contribution ratios. (**b**) The factor loading plots for PC1 vs. PC2. Each plot represents 351 metabolite-candidate peaks, and the loading scores are given in Supplementary Table S3. PC, principal component; MR-CTB51A, MucoRice-CTB transgenic rice line 51A; MR-RNAi, MucoRice-RNAi; NPB-HP, *O*. *sativa* cv. Nipponbare grown hydroponically in a growth chamber; NPB-PF, Nipponbare grown in an open-air paddy field.

Principal component analysis (PCA) was performed with a metabolite data matrix (351 metabolite-candidate peaks versus samples, relative levels as a variable) obtained from the non-polar and polar fractions (Fig. 1). The contribution ratio of principal component 1 (PC1) was 45.8% and that of PC2 was 15.7% (Fig. 1a). The PC1 axis separated the metabolome cluster of MR-CTB51A from those of MR-RNAi, NPB-HP, and NPB-PF, and the PC2 axis likely differentiated the metabolome clusters of transgenic samples (MR-CTB51A and MR-RNAi) from those of non-transgenic samples (NPB-HP and NPB-PF) (Fig. 1a). No clear difference was observed between the metabolome clusters of NPB-HP and NPB-PF (Fig. 1a). The PCA factor loading plot (Fig. 1b) revealed that the separation of metabolome clusters was due not to a few specific metabolites but to several metabolites (Fig. 1b; Supplementary Table S3). These results indicate that the metabolomic profiles of transgenic lines were distinct from those of non-transgenic rice. The metabolomic profile of MR-CTB51A was also different from that of MR-RNAi, suggesting that the expression of modified CTB affected rice seed metabolic activity.

### Comparison of Metabolite Contents in MR-CTB51A, MR-RNAi, and NPB

Among the 351 metabolite-candidate peaks (Supplementary Table S2), 212 peaks were successfully annotated by comparing their retention index values and mass spectra with those of reference compounds (Supplementary Table S4) and 139 peaks remained unannotated (Supplementary Table S5). Among the 212 peaks, 149 were selected as non-redundant metabolites (Supplementary Table S4). Significant differences (*p* < 0.05) in the relative levels of the 149 metabolites are shown in Supplementary Fig. S1.

The relative levels of 85 metabolites differed significantly between MR-CTB51A and NPB-HP (both grown hydroponically in a growth chamber; Table 1). Among them, 82 metabolites (amino acids, organic acids, carbohydrates, and lipids) were present at higher levels in MR-CTB51A, whereas two carbohydrates (d-maltose and sophorose-like 1) and a lipid (β-tocopherol-like 1) were present at lower levels. To evaluate whether growth conditions affected metabolomic profiles, we compared the metabolite levels of NPB-HP and NPB-PF and found significant differences (*p* < 0.05) in the relative levels of 10 metabolites (Supplementary Table S6). We also compared the metabolite levels of MR-CTB51A and NPB-PF and found significant differences (*p* < 0.05) in the relative levels of 76 metabolites (Supplementary Table S7

**Table 1:** The 85 metabolites showing significant differences in relative levels between MR-CTB51A and NPB-HP.

Category^a)^		Metabolite^b)^	Fold difference ^c)^ (MR-CTB51A/NPB-HP)
Amino acids		l-Aspartic acid-like 1	**29.13**
		l-Aspartic acid-like 3	**12.17**
		Ornithine	**9.57**
		l-Lysine	**8.88**
		l-Glutamine	**8.18**
		l-Phenylalanine-like 1	**5.59**
		l-Cysteine	**5.15**
		l-Tryptophan	4.94
		l-Valine	4.58
		Glycine	4.16
		l-Tyrosine	3.99
		l-Isoleucine-like 1	3.89
		l-Leucine	3.63
		Pyroglutamic acid-like 3	3.54
		l-Isoleucine	3.27
		Pyroglutamic acid-like 1^†^	1.75
Amines		Putrescine	**6.72**
Organic acids		l-Malic acid-like 5	**14.00**
		Oxoglutaric acid	**12.92**
		Citric acid	4.64
		Succinic acid-like 3*^†^	2.93
Carbohydrates		d-Ribose-like 3	**6.33**
		d-Glucose	**5.77**
		Glycerol^†^	**5.40**
		Trehalose-like 1	4.47
		Myoinositol	3.00
		Mannitol-like 1	2.61
		Mannitol	2.51
		d-Fructose	2.42
		d-Maltose*^†^	0.53
		Sophorose-like 1^†^	0.45
Nucleic acids		Uridine	3.04
		Adenosine^†^	2.73
Vitamins and cofactors		Ascorbic acid-like 3	2.41
Lipids	Fatty acid methyl esters	C15:0 ME	3.46
		C23:0 ME	3.26
		C21:0 ME	2.68
		C16:1(9*Z*) ME-like 1	2.40
		C20:1(11*Z*) ME*^†^	2.35
		C22:0 ME	2.34
		C22:1(13*Z*) ME-like 1	2.25
		C17:0 ME	2.17
		C20:0 ME	2.16
		C18:0 ME	1.98
		C14:0 ME	1.80
		C16:0 ME-like 1	1.77
		C18:1 ME	1.71
		C18:1 ME-like 1^†^	1.66
		C18:2 ME	1.54
	Fatty acids and conjugates	C19:0-like 1	**6.94**
		C23:0-like 1	**5.49**
		C18:0^†^	**5.39**
		C16:0^†^	4.16
		C22:0	4.15
		C20:0	3.68
		C17:0	3.64
		C15:0	3.45
		C14:0	2.24
		C18:0-like 1	2.20
		C17:0-like 3^†^	2.15
		C21:0-like 1	2.14
		C21:0-like 3	1.97
		C16:0-like 1	1.96
		C13:0-like	1.73
		C18:2-like 1	1.69
	Hydrocarbons	Undecane-like 2	1.79
		Heptadecane-like 1^†^	1.64
		Eicosane^†^	1.60
		Pentadecane^†^	1.44
	Sterols	Cycloartenol	**11.09**
		24-Methylenecycloartanol	**8.70**
		Stigmasterol	3.85
		Cholesterol	3.84
		Campesterol	3.71
		Brassicasterol-like 1^†^	2.86
		β-Sitosterol	2.77
	Prenol lipids	β-Tocopherol	3.04
		Squalene	2.71
		α-Tocopherol	1.72
		β-Tocopherol-like 1	0.43
Others		1-Kestose	**67.90**
		l-3-Cyanoalanine	**10.58**
		Aminoadipic acid	4.62
		Phosphoric acid^†^	2.58
		Phosphoric acid-like 1	2.55

^a)^According to the Kyoto Encyclopedia of Genes and Genomes (KEGG, http://www.genome.jp/kegg/). Lipids were further classified according to the LIPID Metabolites and Pathways Strategy (http://www.lipidmaps.org/). ^b)^Tukey’s test (*p* < 0.05) was used to select metabolites (Supplementary Fig. S1). ME, methyl ester. *Metabolites showing significant difference in relative levels between NPB-HP and NPB-PF (Supplementary Table S6). ^†^Metabolites showing no significant difference in relative levels between MR-CTB51A and NPB-PF (Supplementary Table S7). ^c)^Calculated by dividing the average peak intensity in MR-CTB51A by that in NPB-HP. **Bold numbers**, fold difference >5; MR-CTB51A, MucoRice-CTB transgenic rice line 51A; NPB-HP, *Oryza sativa* cv. Nipponbare grown hydroponically in a growth chamber; NPB-PF, Nipponbare grown in an open-air paddy field.

).

In MR-CTB51A, CTB is expressed in the endosperm and endogenous storage proteins are down-regulated. To investigate the effect of CTB expression as such, we compared MR-CTB51A with the MR-RNAi line carrying an RNAi cassette to down-regulate the endogenous seed storage proteins. The levels of 71 metabolites differed significantly between MR-CTB51A and MR-RNAi (*p* < 0.05): the levels of 65 metabolites (mainly amino acids, organic acids, carbohydrates, and lipids) were higher in MR-CTB51A, and the levels of 6 metabolites [l-tryptophan-like 2, d-fructose-like 2, d-ribose-like 1, glyceric acid, MG(16:0/0:0/0:0), and fructose 6-phosphate-like 1] were lower (Supplementary Table S8). The levels of these 71 metabolites could have been directly or indirectly influenced by CTB expression in the endosperm, but other factors, such as the presence or absence of the selection marker gene in the genome, growth conditions, and the storage period of seeds at room temperature (Supplementary Table S1), should also affect metabolite levels in MR-CTB51A and MR-RNAi.

The levels of 59 metabolites (mainly amino acids and lipids) were significantly higher in MR-CTB51A than in NPB-HP and MR-RNAi (Table 2), including 9 metabolites (ornithine, l-lysine, l-glutamine, putrescine, l-malic acid-like 5, d-ribose-like 3, 24-methylenecycloartanol, 1-kestose, and l

**Table 2:** List of 59 candidate metabolites affected by CTB expression in MR-CTB51A.

Category^a^		Metabolite^b^	Fold difference^c^	
			MR-CTB51A/NPB-HP	MR-CTB51A/MR-RNAi
Amino acids		l-Aspartic acid-like 3	**12**.**17**	2.37
		Ornithine	**9**.**57**	**7**.**10**
		l-Lysine	**8**.**88**	**5**.**22**
		l-Glutamine	**8**.**18**	**32**.**70**
		l-Phenylalanine-like 1	**5**.**59**	4.29
		l-Valine	4.58	3.37
		Glycine	4.16	1.73
		l-Leucine	3.63	2.49
		l-Isoleucine	3.27	1.99
Amines		Putrescine	**6**.**72**	**9**.**25**
Organic acids		l-Malic acid-like 5	**14**.**00**	**11**.**70**
		Oxoglutaric acid	**12**.**92**	3.91
Carbohydrates		d-Ribose-like 3	**6**.**33**	**18**.**13**
		Mannitol-like 1	2.61	**92**.**55**
Lipids	Fatty acid methyl esters	C15:0 ME	3.46	3.16
		C23:0 ME	3.26	2.69
		C21:0 ME	2.68	2.29
		C16:1(9*Z*) ME-like 1	2.40	1.80
		C20:1(11*Z*) ME*^†^	2.35	2.06
		C22:0 ME	2.34	1.95
		C22:1(13*Z*) ME-like 1	2.25	1.70
		C17:0 ME	2.17	2.48
		C20:0 ME	2.16	1.72
		C18:0 ME	1.98	1.68
		C14:0 ME	1.80	1.79
		C16:0 ME-like 1	1.77	1.63
		C18:1 ME	1.71	1.64
		C18:1 ME-like 1^†^	1.66	1.59
		C18:2 ME	1.54	1.60
	Fatty acids and conjugates	C19:0-like 1	**6**.**94**	4.47
		C23:0-like 1	**5**.**49**	2.57
		C22:0	4.15	2.09
		C20:0	3.68	1.78
		C17:0	3.64	2.43
		C15:0	3.45	2.61
		C14:0	2.24	1.78
		C18:0-like 1	2.20	1.56
		C17:0-like 3^†^	2.15	2.07
		C21:0-like 1	2.14	2.43
		C16:0-like 1	1.96	1.56
		C13:0-like 1	1.73	1.66
		C18:2-like 1	1.69	1.66
	Hydrocarbons	Undecane-like 2	1.79	2.26
		Heptadecane-like 1^†^	1.64	1.76
		Eicosane^†^	1.60	1.59
		Pentadecane^†^	1.44	1.63
	Sterols	Cycloartenol	**11**.**09**	3.87
		24-Methylenecycloartanol	**8**.**70**	**6**.**31**
		Stigmasterol	3.85	3.14
		Cholesterol	3.84	2.77
		Campesterol	3.71	2.73
		Brassicasterol-like 1	2.86	2.65
		β-Sitosterol	2.77	2.44
	Prenol lipids	β-Tocopherol	3.04	**5**.**15**
		Squalene	2.71	2.86
		α-Tocopherol	1.72	2.52
Others		1-Kestose	**67**.**90**	**95**.**14**
		l-3-Cyanoalanine	**10**.**58**	**13**.**48**
		Aminoadipic acid	4.62	**5**.**74**

^a)^Categories were assigned as in Table 1. ^b)^Metabolites showing significant differences in relative levels between MR-CTB51A and NPB-HP (Table 1) and between MR-CTB51A and MR-RNAi (Supplementary Table S8) were selected. ME, methyl ester. *Metabolites showing significant difference in relative levels between NPB-HP and NPB-PF (Supplementary Table S6). ^†^Metabolites showing no significant difference in relative levels between MR-CTB51A and NPB-PF (Supplementary Table S7). ^c)^ **Bold numbers**, fold difference >5. MR-RNAi, MucoRice-RNAi; the names of other lines are explained in Table 1.

-3-cyanoalanine) with fold differences of >5 in both comparisons.

The levels of 26 metabolites were significantly different between MR-CTB51A and NPB-HP but not between MR-CTB51A and MR-RNAi and were therefore affected by the downregulation of endogenous seed storage proteins in MR-CTB51A (Table 3). The levels of 23 of these metabolites were higher in MR-CTB51A, including 5 metabolites (l-aspartic acid-like 1, l-cysteine, d-glucose, glycerol, and a C18:0 fatty acid) with fold differences of >5; and 2 carbohydrates (d-maltose and sophorose-like 1) and a lipid (β-tocopherol-like 1) were present at lower levels in MR-CTB51A (Table 3).

### Quantification of γ-Oryzanol Components in MR-CTB51A and NPB

As mentioned above, the relative levels of 24-methylenecycloartanol, a building block for γ-oryzanol, was remarkably higher in MR-CTB51A than in NPB-HP, NPB-PF, and MR-RNAi (Table 1 and Supplementary Fig. S1). γ-Oryzanol is a mixture of steryl ferulates, which are formed by esterification of the 3β-hydroxyl group of sterols (e.g., campesterol, stigmasterol, and β-sitosterol) or triterpene alcohols (e.g., cycloartanol, cycloartenol, 24-methylenecycloartanol, and cyclobranol) with ferulic acid^33, 34^. To determine the γ-oryzanol contents, we extracted total non-esterified lipids from MR-CTB51A, NPB-HP, and NPB-PF and analyzed them by using high-performance liquid chromatography (HPLC) (Supplementary Fig. S2; MR-RNAi was not included in the analysis due to the lack of available samples). The four major chromatographic peaks observed in all samples analyzed were identified as γ-oryzanol components (cycloartenyl ferulate, 24-methylenecycloartanyl ferulate, campesteryl ferulate, and β-sitosteryl ferulate) (Supplementary Fig. S2a). Total γ-oryzanol content (sum of the contents of the four major γ-oryzanol components; μg g^−1^ DW) in MR-CTB51A samples from three independent plants was 756 ± 57.4 (MR-CTB51A-1), 903 ± 15.0 (MR-CTB51A-2), and 761 ± 49.1 (MR-CTB51A-3), i.e., about three times that in the NPB-HP sample (272 ± 48.9) (Supplementary Fig. S2b). The content of cycloartenyl ferulate, 24-methylenecycloartanyl ferulate, and β-sitosteryl ferulate was consistently higher in MR-CTB51A than in NPB-HP (Supplementary Fig. S2c).

Higher levels of γ-oryzanol in MR-CTB51A suggested elevated levels of non-esterified ferulic acid; therefore, we compared the levels of ferulic acid, its biosynthesis precursors (phenylalanine, cinnamic acid, *p*-coumaric acid, caffeic acid), and its metabolite sinapic acid (Supplementary Fig. S3). The relative levels of phenylalanine and cinnamic acid were significantly elevated in MR-CTB51A. The relative level of ferulic acid in MR-CTB51A was not significantly different from that in NPB-HP, but was significantly lower than that in NPB-PF. The relative level of caffeic acid was significantly lower in MR-CTB51A than in NPBs, whereas the *p*-coumaric acid and sinapic acid levels were tended to be elevated in MR-CTB51A.

## Discussion

We found that the seed metabolome of MR-CTB51A was considerably affected by genetic engineering aimed at high CTB expression. Out of 149 metabolites detected in brown rice extracts, the relative levels of 85 metabolites significantly differed between MR-CTB51A and NPB-HP (Table 1). However, 16 out of these 85 metabolites were significantly different between NPB-HP and NPB-PF (Supplementary Table S6) and/or not significantly different between MR-CTB51A and NPB-PF (Supplementary Table S7), suggesting that the contents of the 16 metabolites could be affected by growth conditions (shown in Tables 1–3). The remaining 69 metabolites included amino acids, amines, organic acids, nucleic acids, carbohydrates, vitamins and cofactors, lipids and others; these compounds are candidate metabolites that may have been influenced by genetic engineering aimed at high CTB expression (Table 1). Comparison of the relative levels of the 85 metabolites (Table 1) between MR-CTB51A and MR-RNAi provided us additional information about the possible effects of CTB expression (Table 2) and RNAi-mediated down-regulation of endogenous seed storage proteins (Table 3

**Table 3:** List of 26 candidate metabolites affected by the suppression of seed storage proteins in MR-CTB51A.

Category^a)^		Metabolite^b)^	Fold difference^c)^	
			MR-CTB51A/NPB-HP	MR-CTB51A/MR-RNAi
Amino acids		l-Aspartic acid-like 1	**29**.**13**	1.90
		l-Cysteine	**5**.**15**	1.80
		l-Tryptophan	4.94	0.90
		l-Tyrosine	3.99	0.94
		l-Isoleucine-like 1	3.89	1.24
		Pyroglutamic acid-like 3	3.54	1.61
		Pyroglutamic acid-like 1^†^	1.75	1.51
Organic acids		Citric acid	4.64	1.10
		Succinic acid-like 3*^†^	2.93	1.14
Carbohydrates		d-Glucose	**5**.**77**	3.53
		Glycerol^†^	**5**.**40**	0.73
		Trehalose-like 1	4.47	1.37
		Myoinositol	3.00	1.52
		Mannitol	2.51	1.76
		d-Fructose	2.42	1.73
		d-Maltose*^†^	0.53	1.74
		Sophorose-like 1*^†^	0.45	1.73
Nucleic acids		Uridine	3.04	1.27
		Adenosine^†^	2.73	1.01
Vitamins and cofactors		Ascorbic acid-like 3	2.41	1.47
Lipids	Fatty acids and conjugates	C18:0^†^	**5**.**39**	1.70
		C16:0^†^	4.16	1.92
		C21:0-like 3	1.97	1.26
	Prenol lipids	β-Tocopherol-like 1^†^	0.43	1.08
Others		Phosphoric acid^†^	2.58	0.99
		Phosphoric acid-like 1	2.55	1.21

^a)^Categories were assigned as in Table 1. ^b)^Metabolites showing significant difference in relative levels between MR-CTB51A and NPB-HP (Table 1) but not between MR-CTB51A and MR-RNAi (Supplementary Table S8) were selected. Fatty acids are described as carbon number: unsaturated bond number (e.g., C18:0). *Metabolites showing significant difference in relative levels between NPB-HP and NPB-PF (Supplementary Table S6). ^†^Metabolites showing no significant difference in relative levels between MR-CTB51A and NPB-PF (Supplementary Table S7). ^c)^ **Bold numbers**, fold-difference >5.

) on metabolite levels in MR-CTB51A.

In MR-CTB51A, the CTB protein is expressed and the endogenous seed storage proteins prolamin and glutelin are down-regulated^13, 14^. A substantial portion of the endosperm amino acids is used for the synthesis of storage proteins^35^, and reduced accumulation of glutelins or sulfur-rich 10 kDa prolamin changes total and free amino acid contents in mature rice grains^36^. Therefore, the suppression of endogenous seed storage proteins in the endosperm of MR-CTB51A may primarily affect the amino acid composition and contents in brown rice (Table 1). The level of glutamine, one of the most abundant amino acids in rice prolamin and glutelin (e.g., the glutamine content of the 13-kDa prolamin clone RM1 is 19.2% and that of GluB1 is 11.0%), was remarkably higher in MR-CTB51A than in NPB-HP (Table 1), suggesting a direct relationship between the suppression of endogenous seed storage proteins in the endosperm and amino acid metabolism. The higher levels of two aromatic amino acids (tryptophan and tyrosine) in MR-CTB51A (Table 3) indicate that metabolic flow to the shikimic acid pathway was affected in MR-CTB51A, possibly through the RNAi-mediated suppression of seed storage proteins. The levels of organic acids, carbohydrates, nucleic acids, and lipids also suggest that the genetic engineering might have influenced central metabolism in the seeds of MR-CTB51A (Table 1). Kuroda *et al*.^37^ reported that accumulation of binding protein 1 (BiP1) occurred in the transgenic rice seed harboring β-glucuronidase (GUS)– and green fluorescent protein (GFP)–overexpressing cassettes and RNAi-mediated suppressing cassettes for storage proteins^37^. Cho *et al*.^38^ reported that RNAi-mediated suppression of seed storage proteins in rice grains resulted in accumulation of endoplasmic reticulum (ER) chaperones, such as BiP1 and protein disulfide isomerase-like 1–1 (PDIL1-1)^38^. Proteomic analysis of ER-stressed rice seeds revealed changes in the expression of carbohydrate metabolism–related proteins^39^. It is therefore possible that the down-regulation of prolamin and glutelin has also stimulated ER stress–related responses, thereby influencing carbohydrate metabolism in seeds (Table 3). Lipid metabolism is likely affected in MR-CTB51A, possibly through CTB expression in the endosperm (Table 2). Lipids primarily contributed to the separation of the MR-CTB51A cluster from those of MR-RNAi and NPBs on PC1 (Fig. 1, Supplementary Table S3). Higher levels of fatty acids, prenol lipids, and phytosterols in MR-CTB51A (Table 2) suggest activation of the metabolic pathways that yield acetyl-CoA and pyruvate. The higher levels of fatty acid methyl esters (Table 2) could reflect higher accumulation of glycerolipids, sphingolipids, and other acylated compounds in MR-CTB51A. The levels of these lipids were similar in MR-RNAi and NPBs (Supplementary Fig. S1), indicating that the higher lipid levels in MR-CTB51A could not be ascribed to the down-regulation of seed storage proteins, which is common to MR-CTB51A and MR-RNAi.

Phytosterol levels were also higher in MR-CTB51A than in MR-RNAi and NPBs (Table 1, Supplementary Fig. S1). Total γ-oryzanol content in MR-CTB51A was about three times that in the NPB-HP (272 μg g^−1^ DW; Supplementary Fig. 2b). The γ-oryzanol content in non-pigmented rice (e.g., in the MR-CTB51A parental rice cultivar NPB) is 49 μg g^−1^ DW in the endosperm, 3176 μg g^−1^ DW in bran, 102 μg g^−1^ DW in husk, and 413 μg g^−1^ DW in brown rice^34^. γ-Oryzanol is a generic name of a mixture of phytosterols esterified with different types of monolignols derived from phenylalanine; the levels of the latter were increased in MR-CTB51A (Supplementary Fig. S3). The higher γ-oryzanol levels (Supplementary Fig. S2) could be due to the stimulation of the biosynthesis of phytosterols and shikimic acid pathway metabolites.

Recently, we have developed and established Good Manufacturing Practice-compliant procedures for the generation of the purification-free oral MucoRice-CTB vaccine in a closed production system and have characterized specification and test methods to ensure the quality of the vaccine product^15^. MucoRice-CTB produced in the system was evaluated in a phase I study of MucoRice-CTB conducted with healthy volunteers at a hospital affiliated with the Institute of Medical Science, the University of Tokyo. The dose of MucoRice-CTB orally administered in this study (1.0–6.0 g of powder) is substantially lower than the average amount of daily consumed cooked rice (150 g). Metabolic profiling reported here suggests that the l-3-cyanoalanine content is increased in MR-CTB51A brown rice (Table 1). Toxicity of this compound has been reported in common vetch (*Vicia sativa*) seed^40, 41^; however, l-3-cyanoalanine is naturally produced through the detoxification of cyanide, which is generated as a byproduct during the biosynthesis of the phytohormone ethylene^42^. Because l-3-cyanoalanine is heat stable (95% stable under cooking conditions, i.e., 100 °C for 30 min)^41^, average daily human consumption of cooked non-transgenic brown rice (150 g) should correspond to 21 μg of l-3-cyanoalanine (147 ng g^−1^ dry weight [DW]). We quantified the l-3-cyanoalanine levels in MR-CTB51A and NPBs. The l-3-cyanoalanine content in MR-CTB51A (956 ± 86.0 ng g^−1^ DW) was significantly higher than those in NPB-HP (66.6 ± 1.75 ng g^−1^ DW) and NPB-PF (155 ± 17.4 ng g^−1^ DW). The l-3-cyanoalanine content of the maximum dose of MucoRice-CTB (6 g) is thus estimated to be 5.7 μg. The l-3-cyanoalanine content in MR-CTB51A brown rice (956 ng g^−1^ DW) is lower than that in non-transgenic potato tubers (1.8 μg g^−1^ fresh weight)^26^. No serious adverse responses have been attributed to daily rice consumption^43^.

In summary, we performed GC/TOF-MS–based non-targeted metabolic profiling of brown rice prepared from MR-CTB51A, MR-RNAi, and their non-transgenic parental line, and found, as one might expect, that the MR-CTB51A metabolome was significantly altered by genetic engineering. An increased content of several amino acids, organic acids, carbohydrates, lipids, and secondary metabolites was the most characteristic feature of the seed metabolome in MR-CTB51A. These results provide critical information needed to characterize the impact of genetic engineering on rice metabolic activities. Together with the previous genome sequencing and proteomics studies^14, 16^, this study contributes to the comprehensive understanding of the properties of MucoRice-CTB as a plant-based vaccine.

## Methods

### Plant Materials

The MucoRice-CTB transgenic rice line 51 A (MR-CTB51A)^14^, MucoRice-RNAi (MR-RNAi)^17^, and their parental cultivar NPB were used (Supplementary Table S1). Seedling growth, cultivation, and seed drying and processing were as previously described^15^ with slight modifications. Briefly, MR-CTB51A and NPB seeds were sterilized and soaked in water (pH adjusted to 5.0–5.5) in a growth chamber (MLR-352; Panasonic Healthcare Co., Ltd., Tokyo, Japan) set at 16 h light (26 °C)/8 h dark (24 °C), 300–400 μmol mβ^−2^ s^−1^ light intensity, for 21 days. The seedlings were transferred onto a polystyrene foam board (cultivation panel; M Hydroponic Research Co., Ltd., Aichi, Japan) and were kept for 3 months in a growth chamber at 12 h light (28 °C)/12 h dark (23 °C), 300–400 μmol m^−2^ s^−1^ light intensity. Plants were grown hydroponically using the nutrient solution (OAT house fertilizers 1, 2, and 5; OAT Agrio Co., Ltd., Tokyo, Japan). The NPB seeds were also sown in an open-air paddy field (Niigata, Japan) by conventional method. Rice ears were harvested at least 40 days after heading and dried in a ventilated incubator (MIR-153, Panasonic Healthcare Co.) for several days until water content dropped to approximately 10%. Grains were separated from the ears, and the husks were removed to obtain brown rice, which consists of the seed coat (pericarp and tegument layer), embryo (cotyledon, epicotyl, hypocotyl, and radicle), and endosperm (aleurone cell layer and endosperm cells with starch granules). Brown rice was powdered and stored at −80 °C until use. The MR-RNAi brown rice sample is described in Supplementary Table S1.

### Metabolite Extraction

We prepared five experimental replicates from NPB-HP and MR-RNAi, and four replicates from MR-CTB51A and NPB-PF. Metabolite profiling was performed as reported previously^31, 44^. Briefly, a 50-mg brown rice powder was placed into a 2-mL microtube (Eppendorf AG, Hamburg, Germany) and extracted with 1 mL of a methanol–chloroform–2% acetic acid mixture (5:2:1, v/v/v) containing 5 μg mL^−1^ ribitol and 20 μg mL^−1^ testosterone as internal standards. The suspension was shaken in a mixer mill (Retsch MM400; Verder Scientific Co. Ltd., Haan, Germany) with 300 mg of quartz sand (Wako Pure Chemical Industries, Ltd., Osaka, Japan) for 2 min at a frequency of 30 Hz. The suspension was centrifuged at 12,000 × *g* for 10 min, the supernatant was collected into a new 2-mL microtube (Eppendorf AG), and the precipitate was re-extracted using the same procedure. The supernatants obtained after the first and second extractions were combined. The supernatant (800 μL) was transferred to a new 2-mL microtube (Eppendorf AG), and 500 μL of ultrapure water and 400 μL of chloroform were added and vortexed for 1 min. The sample was centrifuged at 12,000 × *g* for 2 min, the upper phase (polar fraction) was separated from the lower phase collected together with the inter-phase (non-polar fraction); fractions were lyophilized in 2-mL microtubes (Eppendorf AG) in a centrifugal concentrator (CC-105; Tomy Seiko Co. Ltd., Tokyo, Japan) for 6 h (polar fraction) or 1 h (non-polar fraction).

### Preparation of Fatty Acid Methyl Esters

Transesterification of esterified fatty acids to fatty acid methyl esters was performed using a base-catalyzed method. Sodium methoxide (500 μL, approximately 30% in methanol; Sigma-Aldrich Co., St. Louis, MO, USA) was added to the dried non-polar fraction and incubated at 55 °C for 90 min with shaking (1000 rpm; Thermomixer Comfort, Eppendorf AG). Then, 1 mL of 1% acetic acid (to neutralize the NaOH) and 400 μL chloroform were added, and the sample was vortexed and centrifuged at 12,000 × *g* for 2 min. The upper phase was removed, 1 mL of ultrapure water was added and the sample was vortexed and centrifuged again as above. The upper phase was removed, and the remaining lower phase was lyophilized in the same centrifugal concentrator for 1 h.

### Derivatization of Metabolites

Metabolites were derivatized using a combination of methoxamine hydrochloride (Sigma-Aldrich Co.) and *N*-methyl-*N*-(trimethylsilyl)trifluoroacetamide (MSTFA; Sigma-Aldrich Co.). Methoxamine hydrochloride solution (20 μL; 40 mg mL^−1^ in pyridine) was added to the dried polar and non-polar fractions and incubated at 30 °C for 90 min with shaking (1,000 rpm; Thermomixer Comfort). Then, 80 μL of MSTFA was added and incubated at 37 °C for 30 min with shaking as above. The sample was centrifuged at 12,000 × *g* for 3 min, the supernatant was filtered through a hydrophilic polytetrafluoroethylene membrane filter unit (Millex-LG; Merck Millipore Co., Darmstadt, Germany), and a 1-μL aliquot was used in GC/TOF-MS analysis.

### GC/MS Analysis

GC/TOF-MS analysis was performed using a Micromass GCT Premier Mass Spectrometer (Waters Co., Milford, MA, USA) connected to an Agilent 6890 Gas Chromatograph (Agilent Technologies Inc., Santa Clara, CA, USA) and an autosampler (PAL GC-xt; CTC Analytics AG, Zwingen, Switzerland). MassLynx 4.0 software (Waters Co.) was used to control the GC/TOF-MS system. GC was performed using an HP-5ms capillary column, length: 30 m, inner diameter: 0.25 mm, film thickness: 0.25 μm (Agilent Technologies Inc.) at 70 °C for 1 min followed by a 1 °C min^−1^ increase to 76 °C and then 6 °C min^−1^ increase to 350 °C, with a final hold time of 3 min. The carrier gas flow (helium, >99.999% purity; Taiyo Nippon Sanso Co., Tokyo, Japan) was maintained at 1.0 mL min^−1^. Injection port temperature was set at 230 °C. The transfer line temperature between the GC and TOF-MS was 250 °C. Mass spectra were acquired at 10 spectra/s with a mass range of 50–650 *m*/*z*. The mass spectrometer was operated in the positive electron ionization mode. All electron ionization spectra were collected using a detector voltage of 2700 V, electron energy of 70 eV, trap current of 50 μA, filament current of 3.6 A, emission current of 100 μA, and ion source temperature of 250 °C.

### Metabolite Data Handling

MetAlign version 041012^45^ software was used to automatically correct the baseline and to align all extracted mass peaks across all samples with the following parameters. Part A: peak slope factor (× noise) = 4, peak threshold factor (× noise) = 8, peak threshold (absolute value) = 500, average peak width at half height (scans) = 4. Part B: no scaling; internal peak search criteria: beginning of the 1st region = 0 (max. shift = 5); end of the 1st region = 3500 (max. shift = 5); tuning alignment option and criteria: pre-align processing, iterative; calculation criteria for chromatography shift profiles: max. shift per 100 scans = 35; min. factor (× noise) at the 1st and last iterations = 7 each; min. number of masses at the 1st and last iterations = 10 and 5, respectively. The processed data was imported into AIoutput2 version 1.30^30^ (http://prime.psc.riken.jp/Metabolomics_Software/AIoutput/index.html) to deconvolute ion peaks to metabolite-candidate peaks, to convert retention time to retention index values, and to annotate metabolite-candidate peaks using an in-house mass spectral library with the following parameters: height threshold = 500; RT bining = 1; available index, retention index; analysis type, non-targeted; RI tolerance = 20; match threshold (identification score) = 0.6; filtering, accurate; height filter = 3000; RSD (CV) filter = 30. Peak intensity ratios of metabolites and internal standards in each TIC chromatogram were used to compare metabolite levels among different samples. Testosterone and ribitol were used as internal standards for non-polar and polar fractions, respectively.

A total of 351 metabolite-candidate peaks were annotated (Supplementary Table S2): 198 (N001–N198) from the non-polar fraction and 153 (P001–P153) from the polar fraction. Peaks were identified on the basis of Pearson’s product–moment correlation coefficient (PPMCC) considering the retention time and weighted mass spectrum of each metabolite-candidate peak (Supplementary Table S2) and those of the compounds in our reference library; 212 peaks that matched compounds in the library were called annotated peaks, and 139 peaks remained unannotated. The annotated peaks were listed with the library compound name and the values of the identification score, Delta-RI, and PPMCC (Supplementary Table S4); Delta-RI was obtained by subtracting the retention index value of a metabolite-candidate peak from that of a library compound. When an annotated peak corresponded to a single top hit compound, the peak was tentatively given the name of this compound (if the identification score was ≥0.800) or the name of this compound followed by “-like 1” (if the identification score was <0.800). When at least two annotated peaks corresponded to the same top hit compound, the name of this compound was tentatively assigned to the peak with the highest identification score (≥0.800), and the name of this compound followed by “-like 1, -like 2, …” was assigned to the remaining peaks in order of decreasing identification scores. If the identification score was <0.800, the name of the top hit compound with “-like 1, -like 2, …” was assigned in order of decreasing identification scores. Potentially redundant peaks were excluded from in-depth evaluation of metabolomic profiles. When multiple peaks had the same top hit compound name and showed similar accumulation profiles, a single peak with the highest identification score was selected for further evaluation (Supplementary Table S4) so that selected peaks represented different metabolites. The tentative metabolite name of each selected peak was used throughout this study. In the case of l-threonine-like 1 and l-threonine-like 2, only the peak with the second highest identification score (l-threonine-like 2) was selected because the peak with the highest identification score (l-threonine-like 1) did not show good reproducibility in relative levels among experimental replicates.

### Total Lipid Extraction

Total lipids were extracted from brown rice powder by using the Folch method^46^. Briefly, samples (50 mg) were extracted with 1 mL of a chloroform–methanol mixture (2:1, v/v) at room temperature by shaking in a mixer mill (Retsch MM400) with 300 mg of quartz sand (Wako Pure Chemical Industries, Ltd.) for 2 min at a frequency of 30 Hz and then stirred on a rotator (25 rpm; RT-50, Taitec Co., Saitama, Japan) at room temperature for 20 min. The mixture was centrifuged at 12,000 × *g* for 3 min and the supernatant was transferred to a new 2-mL microtube (Eppendorf AG). Extraction was repeated with 0.5 mL of the extraction solvent, and the supernatants were combined. Sodium chloride (0.3 mL; 0.9% in ultrapure water; Nacalai Tesque Inc., Kyoto, Japan) was then added, mixed by vortexing, and the sample was centrifuged at 12,000 × *g* for 3 min. The upper phase was removed and the remaining lower phase was lyophilized in a centrifugal concentrator (CC-105) for 1 h. Methanol (300 μL) was added to the lyophilized lipid extract, and the sample was vortexed, sonicated, and centrifuged at 12,000 × *g* for 3 min. The supernatant was filtered through a hydrophilic polytetrafluoroethylene membrane filter unit (Millex-LG). An aliquot of the filtrate (5 μL) was used for HPLC analysis to determine the γ-oryzanol content.

### HPLC Analysis

HPLC analysis was performed using a LaChrom Elite system (pump, L-2130; auto sampler, L-2200; column oven, L-2300; photo diode array detector, L-2455; Hitachi High-Technologies Co., Tokyo, Japan). Metabolites were separated on a Cadenza CD-C18 column (length, 150 mm; internal diameter, 2.0 mm; particle size, 3 μm; Imtakt Co., Kyoto, Japan) by isocratic elution in a methanol–acetonitrile–acetic acid mixture (52:45:3, v/v/v)^47^, at a column temperature of 40 °C, a flow rate of 0.2 mL min^−1^ and detection at 315 nm. γ-Oryzanol components were quantified by external calibration on the basis of elution time and the ultraviolet–visible absorbance spectra of authentic standard compounds (Wako Pure Chemical Industries, Ltd.).

### Statistical Analyses

Principal component analysis (PCA) and one-way analysis of variance (ANOVA) with Tukey’s honestly significant difference (HSD) post hoc test were performed with the web-based free software MetaboAnalyst 3.0^48^. Data scaling for PCA was auto scaling (mean-centered and divided by the standard deviation of each variable). The Student’s *t*-test was performed using Microsoft Excel for Mac 2011 (Microsoft Co., Redmond, WA, USA).

### Analysis of Metabolites in Phenylpropanoid Biosynthesis Pathway

Brown rice samples were powdered and extracted with a methanol–chloroform–acetic acid mixture. The obtained polar fractions were derivatized using a combination of methoxamine hydrochloride and MSTFA, and 1-μL aliquots were subjected to GC/TOF-MS analyses. Identification of metabolites was based on the mass spectrum and retention index matching to those of authentic standard compounds. Peak area ratios of the metabolites and the internal standard (ribitol) in each TIC chromatogram were used to compare metabolite levels among different samples.

### Data Availability

All raw data and associated metadata generated during the current study are available in MetaboLights^49^ (http://www.ebi.ac.uk/metabolights/), an open access data repository, with an ID of MTBLS437.

## Electronic supplementary material

Supplementary Information

Supplementary Tables Dataset
