# Supplementary material for: Seed Metabolome Analysis of a Transgenic Rice Line Expressing Cholera Toxin B-subunit
Source: Sci Rep. 2017 Jul 12;7:5196. doi: 10.1038/s41598-017-04701-w (PMC5507873; doi:10.1038/s41598-017-04701-w)
Supplement: Supplementary file 1 — Supplementary Information [file 41598_2017_4701_MOESM1_ESM.pdf]

Supplementary Information:

Seed Metabolome Analysis of a Transgenic Rice Line Expressing Cholera Toxin B-subunit

Takumi Ogawa[1], Koji Kashima[2], Yoshikazu Yuki[2,3], Mio Mejima[2], Shiho Kurokawa[2], Masaharu Kuroda[4], Atsushi Okazawa[1], Hiroshi Kiyono[2,3], Daisaku Ohta[1]*

[1] Laboratory of Cell Metabolism and Function, Division of Molecular Biology and Cell Informatics, Department of Applied Life Sciences, Graduate School of Life and Environmental Sciences, Osaka Prefecture University, Osaka 599-8531, Japan

[2] Tokyo Mucosal Patches Laboratory, Division of Mucosal Immunology, Department of Microbiology and Immunology, The Institute of Medical Science, The University of Tokyo, Tokyo 108-8639, Japan

[3] International Research and Development Center for Mucosal Vaccines, The Institute of Medical Science, The University of Tokyo, Tokyo 108-8639, Japan

[4] Crop Development Division, NARO Agriculture Research Center, Niigata 943-0193 Japan

*ohtad2g30490@bioinfo.osakafu-u.ac.jp

Supplementary Figures

Supplementary Figures (pp. 3−11)

Supplementary Figure S1. Relative levels of 149 selected metabolites.
Supplementary Figure S2. Analysis of γ-oryzanol components in brown rice seeds.
Supplementary Figure S3. Relative levels of phenylpropanoids.

Supplementary Table Information (pp. 12)

Following Tables are attached as Excel spreadseets.

Supplementary Table S1. Brown rice samples used in this study.
Supplementary Table S2. List of 351 metabolite-candidate peaks detected in brown rice extracts.
Supplementary Table S3. Factor loadings from principal component analysis shown in Figure 1B.
Supplementary Table S4. Peak identification and selection.
Supplementary Table S5. Comparisons of the relative levels of the 139 unannotated metabolite-candidate peaks.
Supplementary Table S6. Metabolites showing significant differences in relative levels between NPB-HP and NPB-PF.
Supplementary Table S7. Metabolites showing significant differences in relative levels between MR-CTB51A and NPB-PF.
Supplementary Table S8. Metabolites showing significant differences in relative levels between MR-CTB51A and MR-RNAi.

1. Peptides

1-1. Amino acids

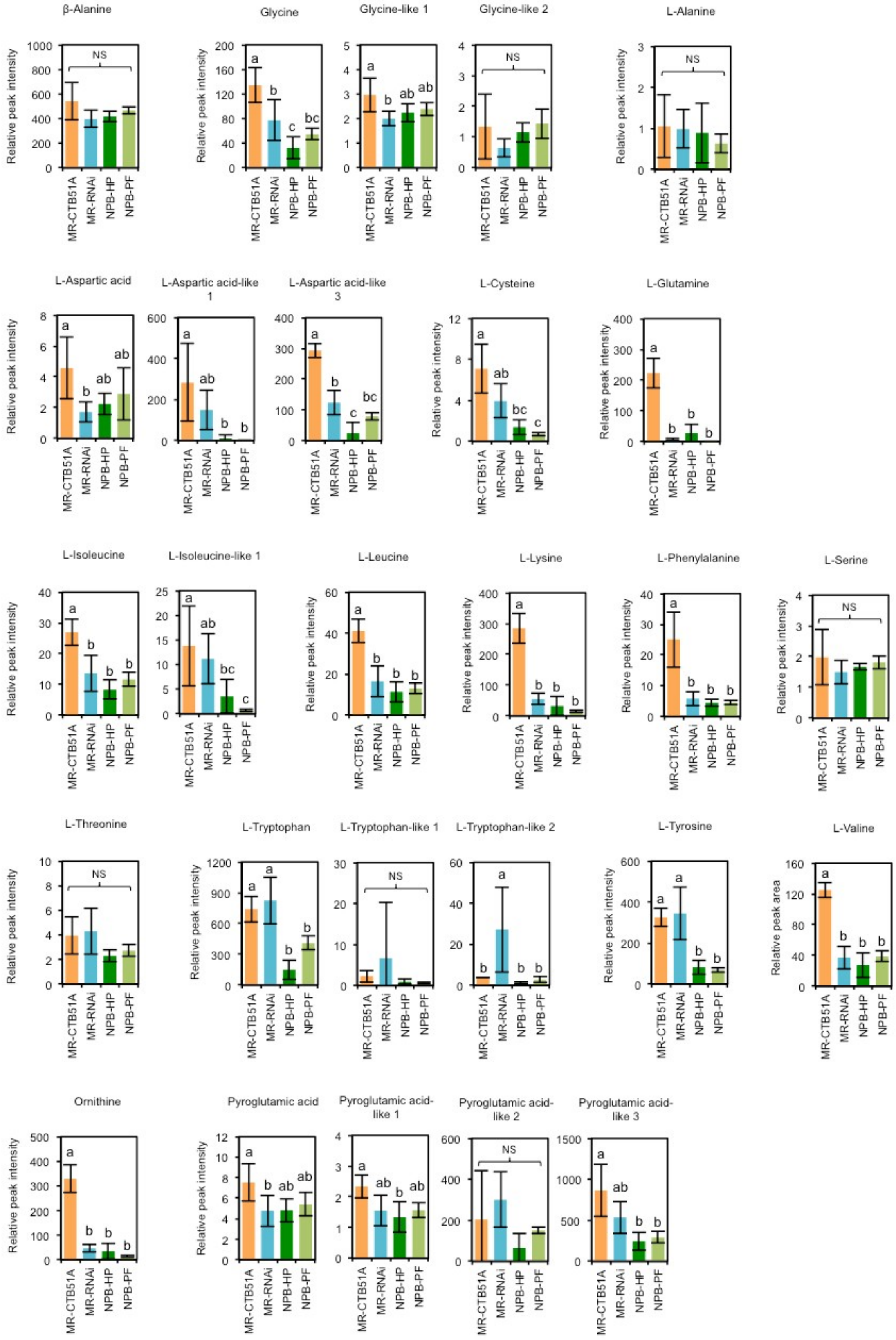

1. Peptides

1-2. Amines

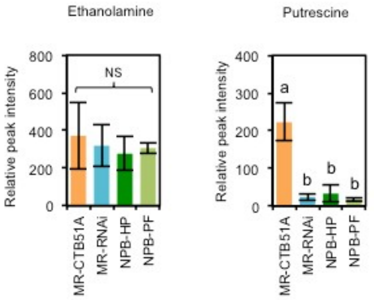

2. Organic acids

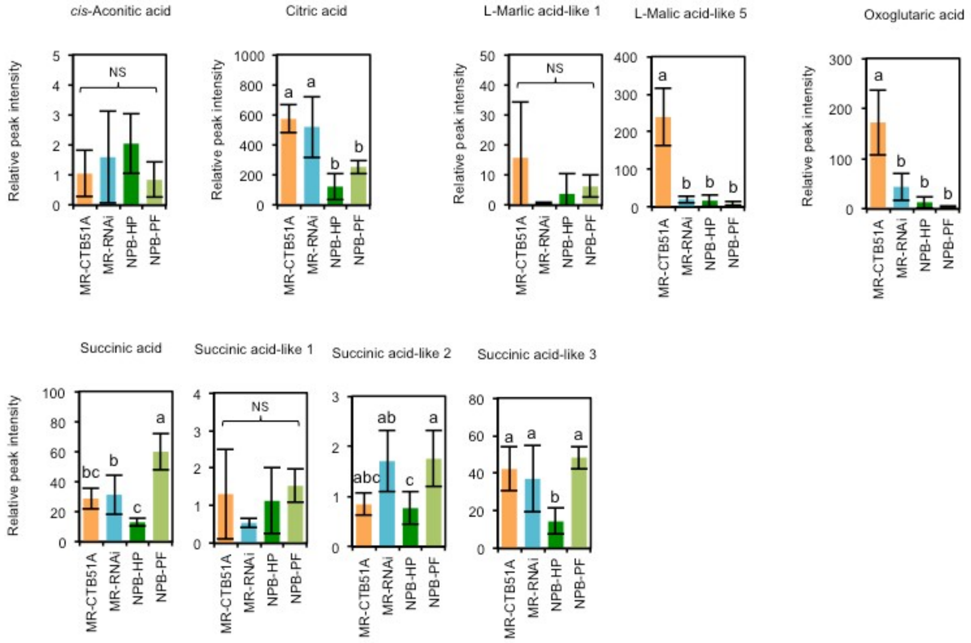

3. Nucleic acids

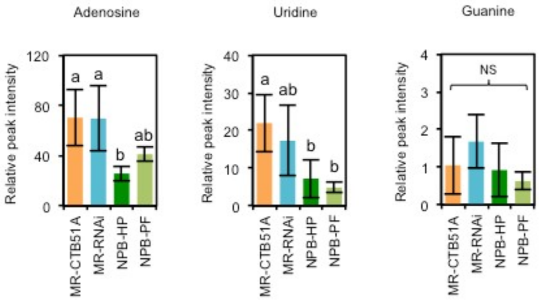

4. Vitamins and cofactors

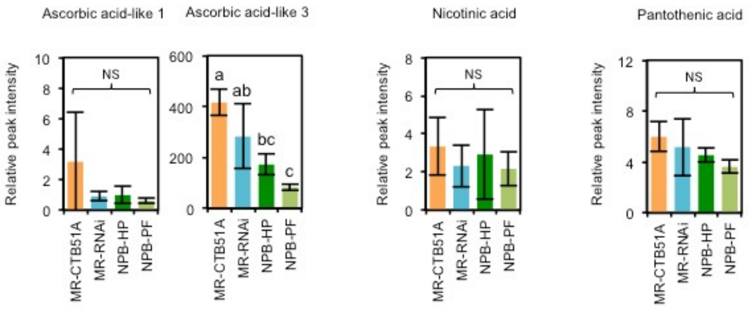

5. Carbohydrates

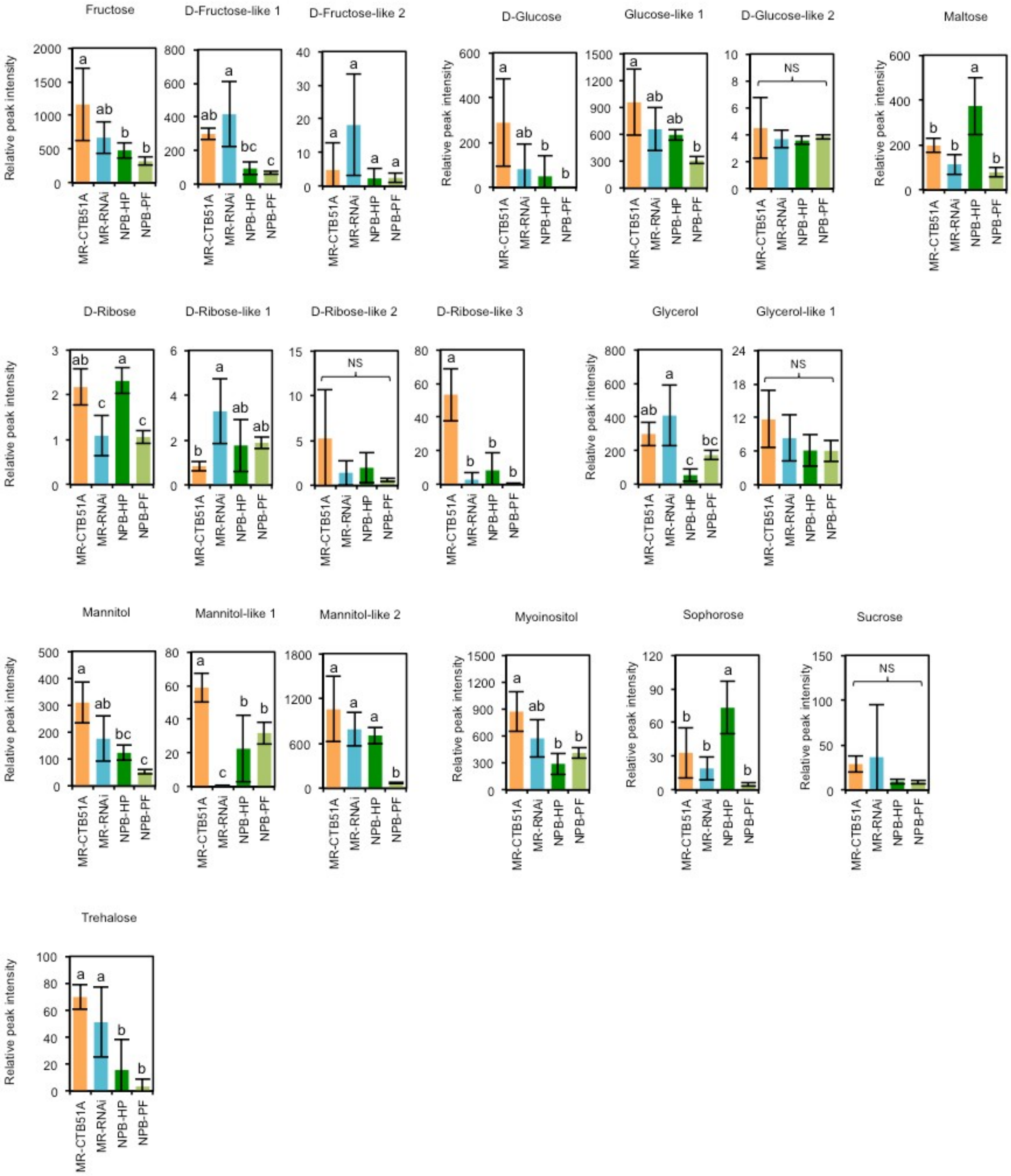

6. Lipids

6-1. Fatty acid methyl esters

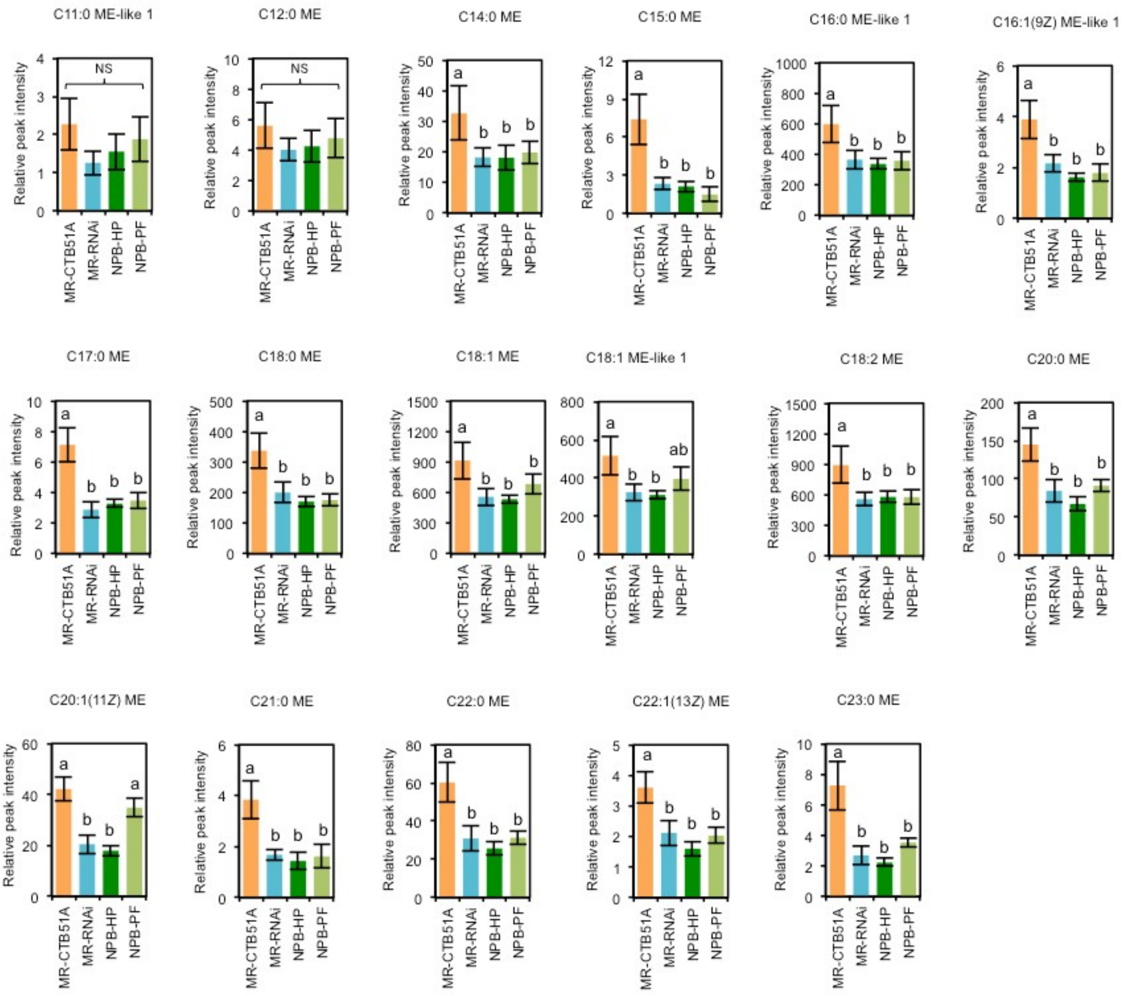

6-2. Fatty acyls (Fatty acids and conjugates)

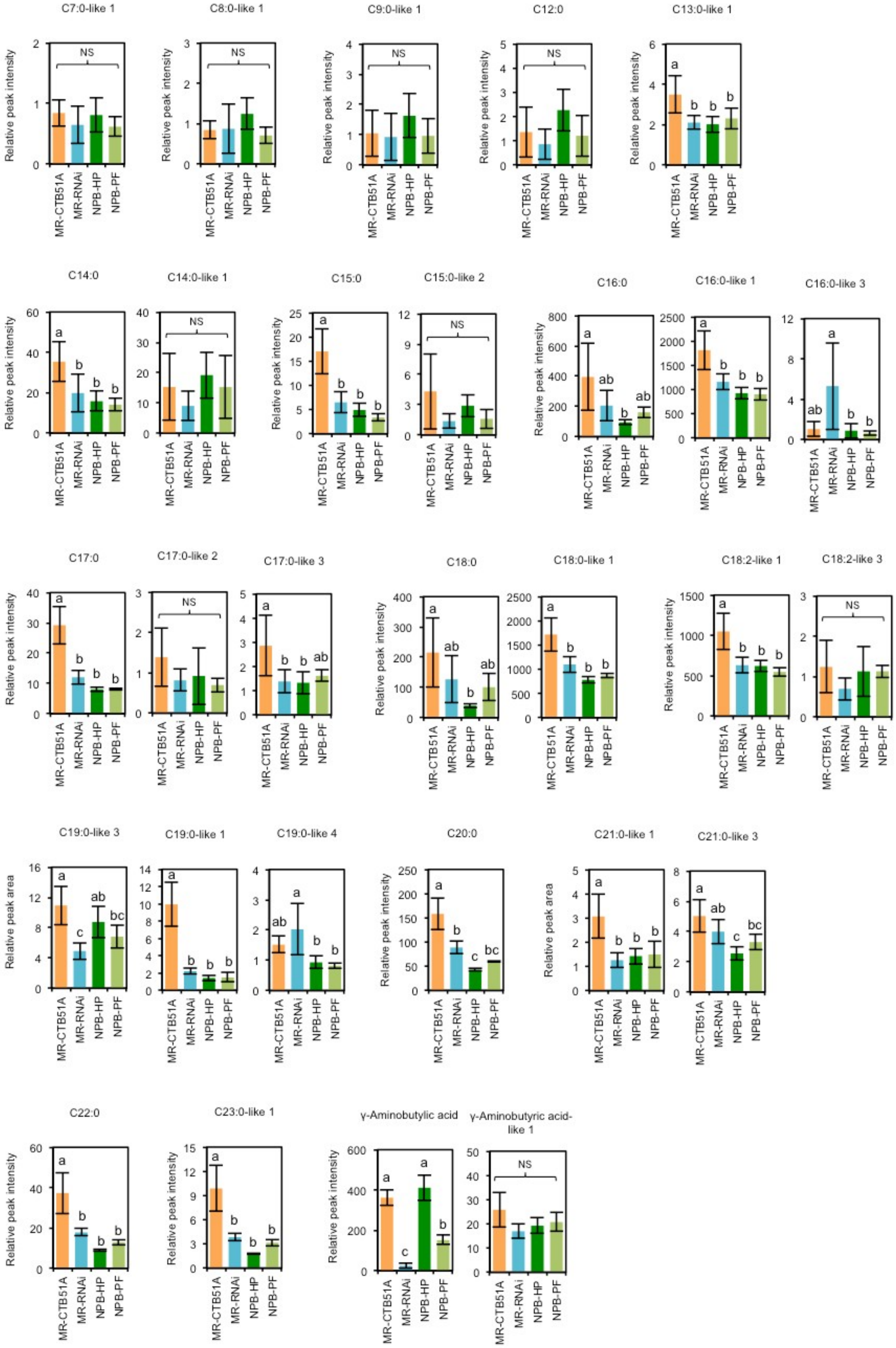

6-2. Fatty acyls (Hydrocarbons)

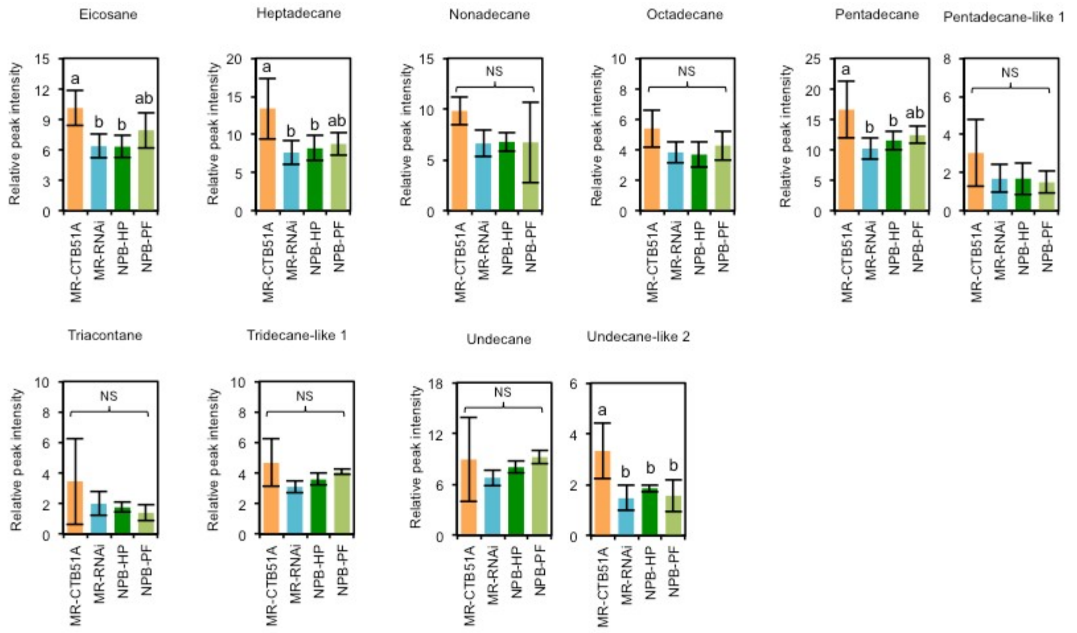

6-3. Sterols

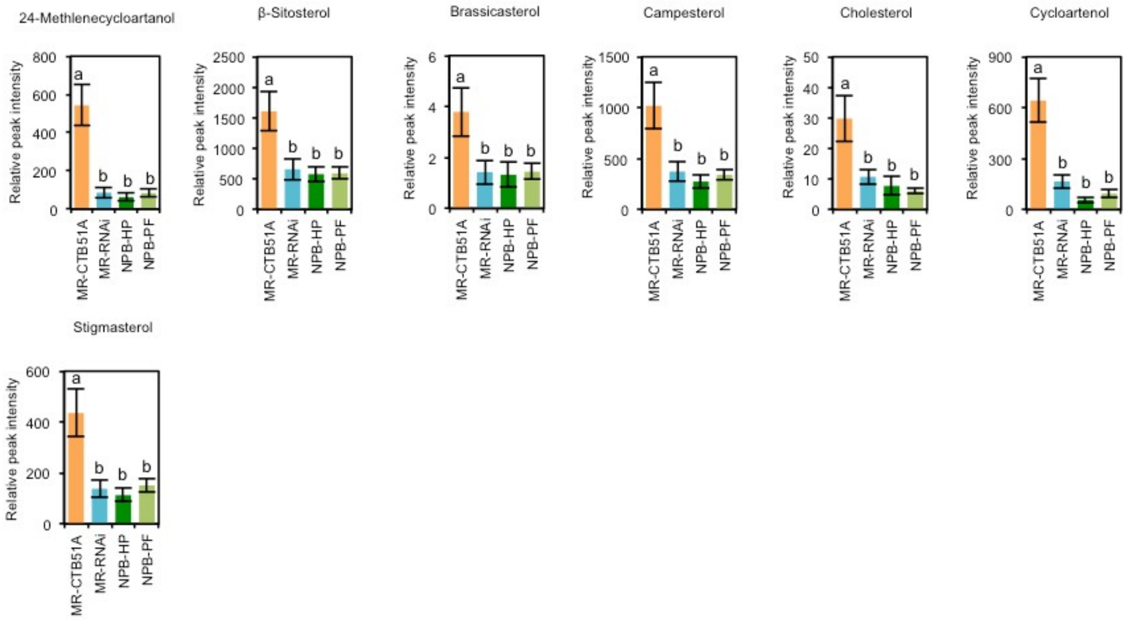

6-4. Prenol lipids

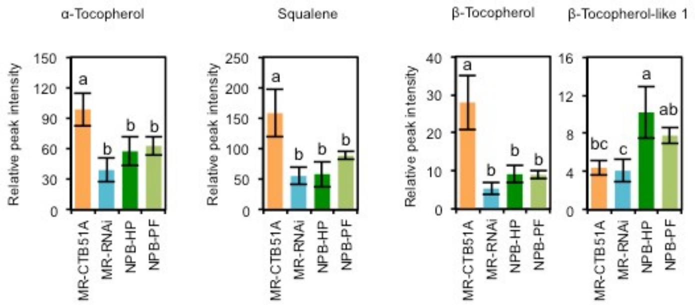

7. Others

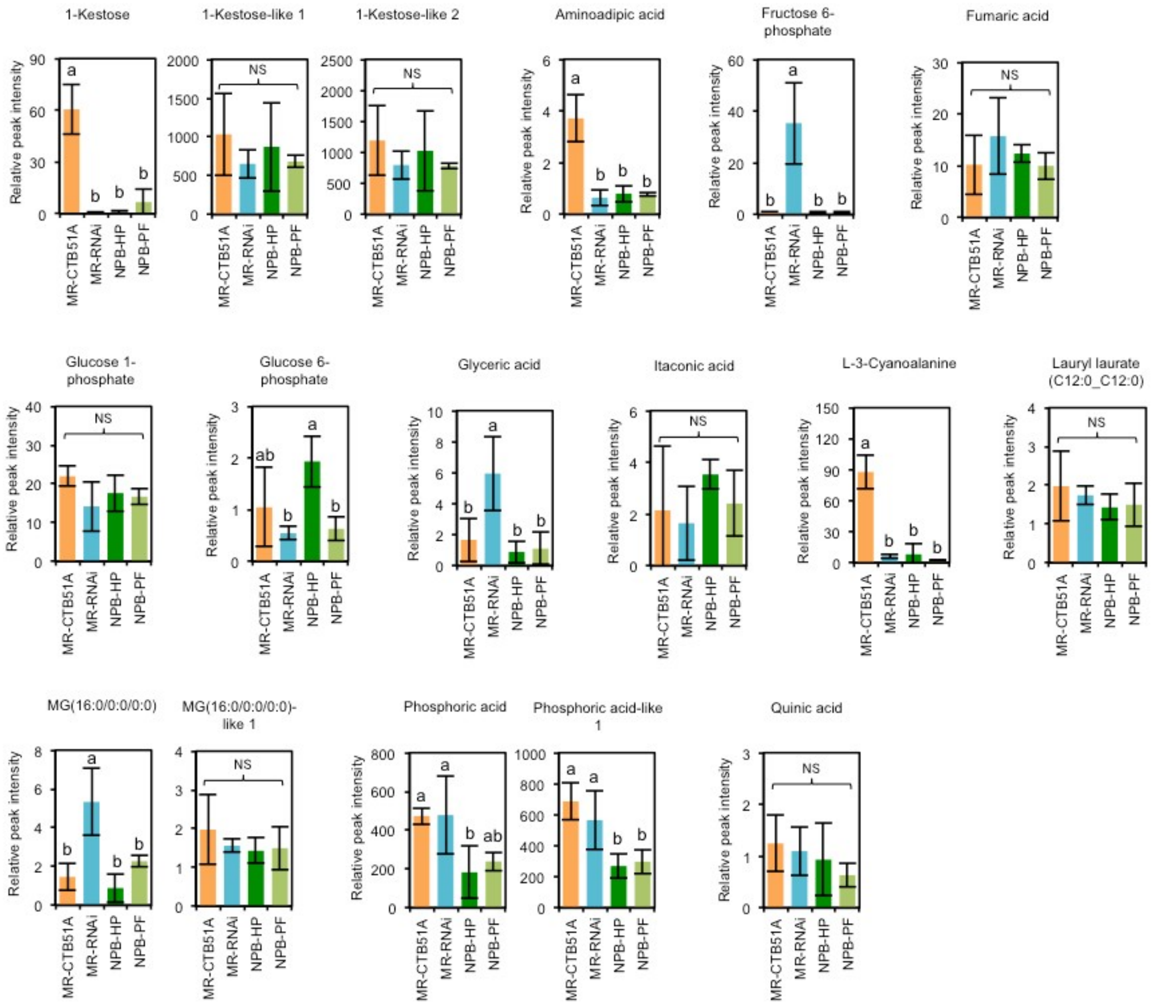

Supplementary Figure S1. Relative levels of 149 selected metabolites.

Values are average ± standard deviation (*n* = 5, NPB-HP and MR-RNAi; *n* = 4, MR-CTB51A and NPB-PF; experimental replicates). Metabolite categories were classified according to the Kyoto Encyclopedia of Genes and Genomes (KEGG, http://www.genome.jp/kegg/). Lipids were further classified following LIPID Metabolites and Pathways Strategy (http://www.lipidmaps.org/). Metabolite names are as in Supplementary Table S4. Different lower-case letters indicate significant differences ($p < 0.05$) in one-way analysis of variance with Tukey's honestly significant difference post hoc test. NS, not significant. MR-CTB51A, MucoRice-CTB transgenic rice line 51A; MR-RNAi, MucoRice-RNAi; NPB-HP, *Oryza sativa* cv. Nipponbare grown hydroponically in a growth chamber; NPB-PF, Nipponbare grown in an open-air paddy field.

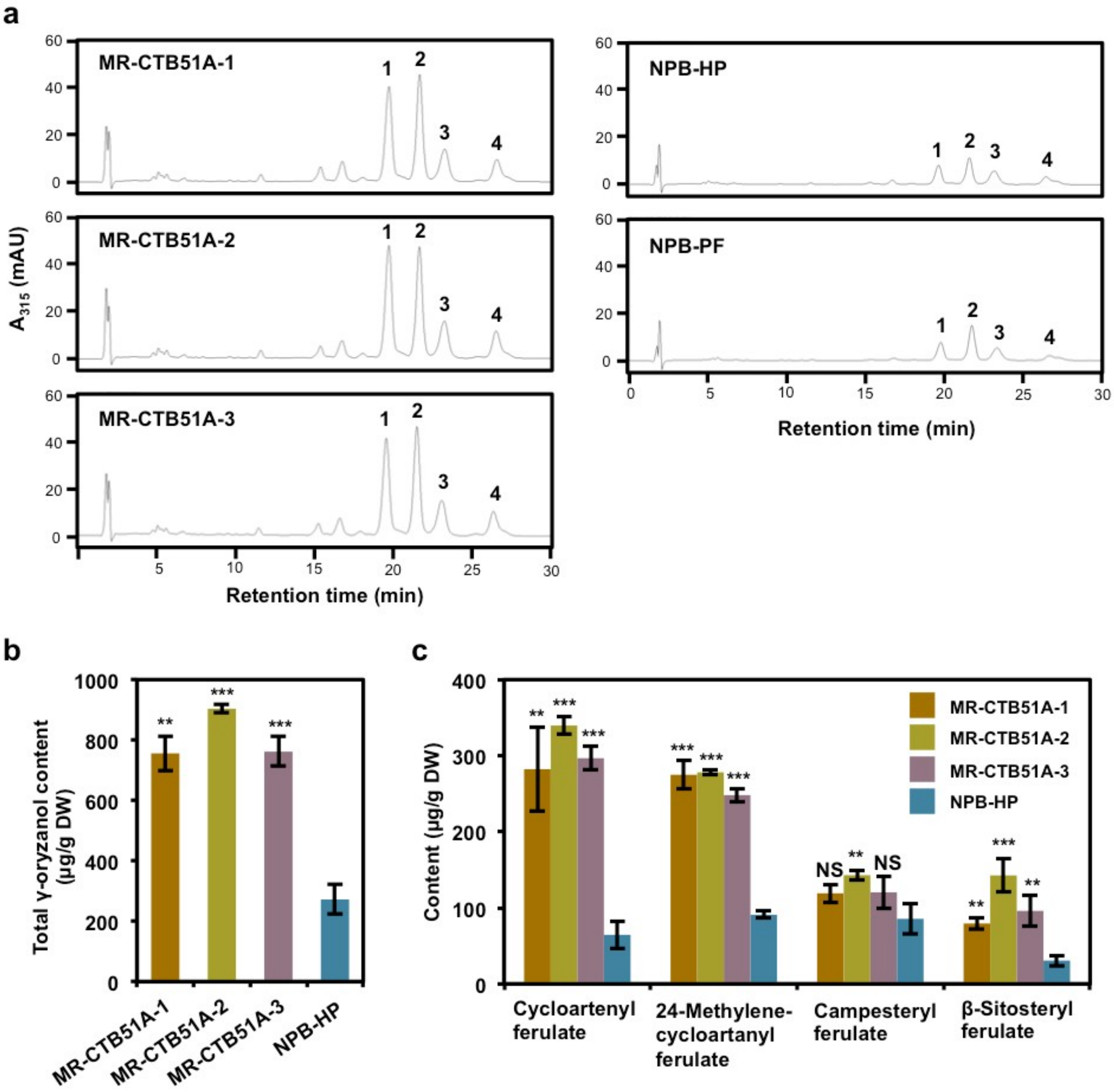

Supplementary Figure S2. Analysis of γ-oryzanol components in brown rice seeds.

(a) HPLC chromatograms (detection at 315 nm). Chromatographic peaks were identified as follows: 1, cycloartenyl ferulate; 2, 24-methylenecycloartanyl ferulate; 3, campesteryl ferulate; and 4, β-sitosteryl ferulate. (b) Total γ-oryzanol content. Values are mean ± standard deviation (n = 3, experimental replicates). (c) Content of four major γ-oryzanol components. Values are mean ± standard deviation (n = 3, experimental replicates). Rice line names are explained in Supplementary Fig. S1. SD, standard deviation. In (b) and (c), asterisks indicate statistically significant differences (Student's t-test with Bonferroni correction, MR-CTB51A vs. NPB-HP; ** $p < 0.01$, *** $p < 0.001$; NS, not significant). DW, dry weight.

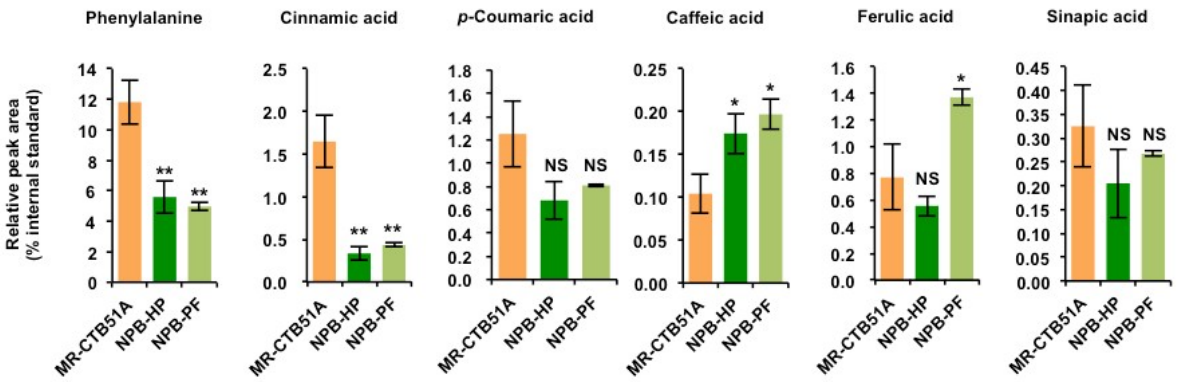

Supplementary Figure S3. Relative levels of phenylpropanoids. Values are mean ± standard deviation (*n* = 3, experimental replicates). Asterisks indicate statistically significant differences in relative levels between MR-CTB51A and other samples (Student's *t*-test with Bonferroni correction for multiple comparisons; *$p < 0.05$, **$p < 0.01$; NS, not significant). Rice line names are explained in Supplementary Fig. S1.

Supplementary Table Information (Attached as Excel spreadseets)

Supplementary Table S1. Brown rice samples used in this study.
Supplementary Table S2. List of 351 metabolite-candidate peaks detected in brown rice extracts.
Supplementary Table S3. Factor loadings from principal component analysis shown in Figure 1B.
Supplementary Table S4. Peak identification and selection.
Supplementary Table S5. Comparisons of the relative levels of the 139 unannotated metabolite-candidate peaks.
Supplementary Table S6. Metabolites showing significant differences in relative levels between NPB-HP and NPB-PF.
Supplementary Table S7. Metabolites showing significant differences in relative levels between MR-CTB51A and NPB-PF.
Supplementary Table S8. Metabolites showing significant differences in relative levels between MR-CTB51A and MR-RNAi.
